# Whole exome sequencing and machine learning germline analysis of individuals presenting with extreme phenotypes of high and low risk of developing tobacco-associated lung adenocarcinoma

**DOI:** 10.1016/j.ebiom.2024.105048

**Published:** 2024-03-13

**Authors:** Ana Patiño-García, Elizabeth Guruceaga, Maria Pilar Andueza, Marimar Ocón, Jafait Junior Fodop Sokoudjou, Nicolás de Villalonga Zornoza, Gorka Alkorta-Aranburu, Ibon Tamayo Uria, Alfonso Gurpide, Carlos Camps, Eloísa Jantus-Lewintre, Maria Navamuel-Andueza, Miguel F. Sanmamed, Ignacio Melero, Mohamed Elgendy, Juan Pablo Fusco, Javier J. Zulueta, Juan P. de-Torres, Gorka Bastarrika, Luis Seijo, Ruben Pio, Luis M. Montuenga, Mikel Hernáez, Idoia Ochoa, Jose Luis Perez-Gracia

**Affiliations:** a Department of Pediatrics and Clinical Genetics, Clínica Universidad de Navarra (CUN), Cancer Center Clínica Universidad de Navarra (CCUN), Program in Solid Tumors, Center for Applied Medical Research (Cima) and Navarra Institute for Health Research (IdisNA), University of Navarra, Pamplona, Spain; b Bioinformatics Platform, Cima and IdisNA, University of Navarra, Pamplona, Spain; c Department of Oncology, CUN, CCUN and IdisNA, University of Navarra, Pamplona, Spain; d Pulmonary Department, CUN, CCUN and IdisNA, University of Navarra, Pamplona, Spain; e Electrical and Electronic Engineering Department, Tecnun, University of Navarra, San Sebastian, Spain; f CIMA LAB Diagnostics and IdisNA, University of Navarra, Pamplona, Spain; g Department of Medical Oncology, Hospital General Universitario de Valencia, Unidad Mixta TRIAL (Fundación para la Investigación del Hospital General Universitario de Valencia y Centro de Investigación Príncipe Felipe) and Centro de Investigación Biomédica en Red Cáncer (CIBERONC), Valencia, Spain; h Department of Biotechnology, Universitat Politècnica de València, Unidad Mixta TRIAL (Fundación para la Investigación del Hospital General Universitario de Valencia y Centro de Investigación Príncipe Felipe) and CIBERONC, Valencia, Spain; i Department of Oncology, CUN, Division of Immunology, Cima, CCUN, IdisNA and CIBERONC, University of Navarra, Pamplona, Spain; j Division of Immunology, Cima and Immunotherapy, CUN, CCUN, IdisNA and CIBERONC, University of Navarra, Pamplona, Spain; k Institute for Clinical Chemistry and Laboratory Medicine, Mildred-Scheel Early Career Center, National Center for Tumor Diseases Dresden (NCT/UCC), University Hospital and Faculty of Medicine, Medical Clinic I, University Hospital Carl Gustav Carus, Technische Universität Dresden, Dresden, Germany. Laboratory of Cancer Cell Biology, Institute of Molecular Genetics of the Czech Academy of Sciences, Prague, Czech Republic; l Department of Medical Oncology Hospital La Luz, Quirón, Madrid, Spain; m Pulmonary, Critical Care, and Sleep Division, Mount Sinai Morningside Hospital, New York, USA; n Department of Radiology, CUN, CCUN and IdisNA, Pamplona, Spain; o Pulmonary Department, CUN, CCUN and Centro de Investigación Biomédica en Red de Enfermedades Respiratorias (CIBERES), University of Navarra, Madrid, Spain; p Program in Solid Tumors, Cima -CCUN, Department of Biochemistry and Genetics, School of Science, IdisNA and CIBERONC, University of Navarra, Pamplona, Spain; q Program in Solid Tumors, Cima, Department of Pathology, Anatomy and Physiology, Schools of Medicine and Sciences, CCUN, IdisNA and CIBERONC, University of Navarra, Pamplona, Spain; r Computational Biology Program, Cima, Data Science and Artificial Intelligence Institute (DATAI), CCUN, IdisNA and CIBERONC, University of Navarra, Pamplona, Spain; s Electrical and Electronic Engineering Department, Tecnun, DATAI, University of Navarra, San Sebastian, Spain; t Department of Oncology, CUN, CCUN, IdisNA and CIBERONC, University of Navarra, Pamplona, Spain

**Keywords:** Cancer risk, Extreme phenotypes, Whole exome sequencing, Tobacco, Lung adenocarcinoma

## Abstract

**Background:**

Tobacco is the main risk factor for developing lung cancer. Yet, while some heavy smokers develop lung cancer at a young age, other heavy smokers never develop it, even at an advanced age, suggesting a remarkable variability in the individual susceptibility to the carcinogenic effects of tobacco. We characterized the germline profile of subjects presenting these extreme phenotypes with Whole Exome Sequencing (WES) and Machine Learning (ML).

**Methods:**

We sequenced germline DNA from heavy smokers who either developed lung adenocarcinoma at an early age (*extreme cases*) or who did not develop lung cancer at an advanced age (*extreme controls*), selected from databases including over 6600 subjects. We selected individual coding genetic variants and variant-rich genes showing a significantly different distribution between extreme cases and controls. We validated the results from our discovery cohort, in which we analysed by WES extreme cases and controls presenting similar phenotypes. We developed ML models using both cohorts.

**Findings:**

Mean age for extreme cases and controls was 50.7 and 79.1 years respectively, and mean tobacco consumption was 34.6 and 62.3 pack-years. We validated 16 individual variants and 33 variant-rich genes. The gene harbouring the most validated variants was *HLA-A* in extreme controls (4 variants in the discovery cohort, p = 3.46E-07; and 4 in the validation cohort, p = 1.67E-06). We trained ML models using as input the 16 individual variants in the discovery cohort and tested them on the validation cohort, obtaining an accuracy of 76.5% and an AUC-ROC of 83.6%. Functions of validated genes included candidate oncogenes, tumour-suppressors, DNA repair, HLA-mediated antigen presentation and regulation of proliferation, apoptosis, inflammation and immune response.

**Interpretation:**

Individuals presenting extreme phenotypes of high and low risk of developing tobacco-associated lung adenocarcinoma show different germline profiles. Our strategy may allow the identification of high-risk subjects and the development of new therapeutic approaches.

**Funding:**

See a detailed list of funding bodies in the Acknowledgements section at the end of the manuscript.

**Research in context**

**Evidence before this study**

To our knowledge, no prior studies have validated the genomic profile of individuals presenting with extreme phenotypes of high and low risk of developing tobacco-associated lung adenocarcinoma.

**Added value of this study**

Our findings support that individuals presenting these clinically relevant phenotypes, which likely represent real life models of extreme sensitivity and resistance to the carcinogenic effects of tobacco, show different germline profiles. Validated genes are involved in functions relevant for cancer development.

**Implications of all the available evidence**

Our strategy may allow for the identification of individuals presenting high-risk of developing tobacco-associated lung adenocarcinoma and may lead to the development of new preventive and therapeutic approaches.

## Introduction

Lung cancer is the tumour responsible for the largest number of deaths worldwide,^1^ and its main risk factor is tobacco.^2^^,^^3^ Yet, while some heavy smokers never develop lung cancer, even at an advanced age, other heavy smokers develop the disease at a young age, suggesting that the susceptibility to developing lung cancer associated with tobacco consumption is not uniform, and that large interindividual differences exist for this association. These clinically relevant phenotypes likely represent real life models of extreme sensitivity and resistance to the carcinogenic effects of tobacco. The identification of the underlying biological causes could allow the identification of high-risk populations, which could benefit from prevention and screening programs; and could lead to new preventive and therapeutic developments for this disease.

Several risk prediction models for lung cancer have been developed,^4^^,^^5^ some of which include genetic variants. Nevertheless, none considers either the clinical or the genomic information that could be provided by individuals presenting these extreme phenotypes of high and low susceptibility to developing tobacco-associated lung cancer.

We previously characterized with Whole Exome Sequencing (WES) the germline genetic profile of individuals presenting extreme phenotypes of very high and very low susceptibility to developing tobacco-associated lung adenocarcinoma, i.e.,: heavy smokers who either developed lung adenocarcinoma at a young age (*extreme cases*, n = 50), or who did not develop lung cancer at an advanced age (*extreme controls*, n = 50).^6^ We limited the selection of extreme cases to lung adenocarcinoma to increase sample homogeneity. We observed significant differences among both groups in variants located in genes related to maintenance of genomic stability, regulation of inflammation, detoxification, transcriptional regulation, and HLA-mediated antigen presentation, among others.

Here, we validate those results in a larger and independent cohort of individuals presenting extreme phenotypes of similar characteristics, using WES and Machine Learning (ML) analysis.

## Methods

### Design

**Fig. 1:**
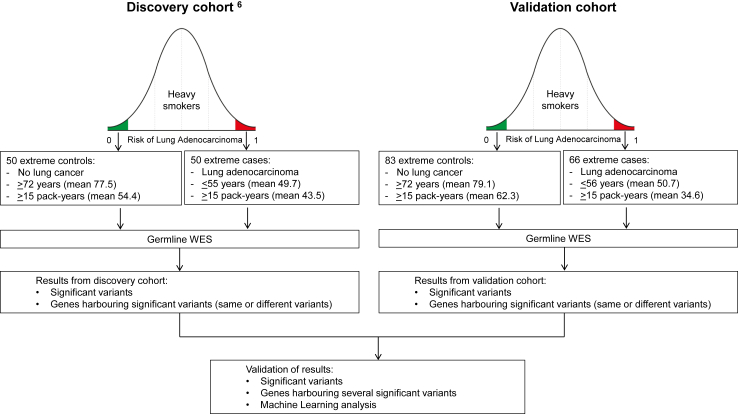
**Study design.** We hypothesized that the risk of developing tobacco-associated lung adenocarcinoma is not uniform, and that genetic profiles potentially associated with increased and decreased susceptibility to developing tobacco-associated lung adenocarcinoma would be enriched in these populations. WES: Whole Exome Sequencing.

We characterized with germline WES the genotype of individuals presenting extreme phenotypes of very high and very low risk of developing tobacco-associated lung adenocarcinoma and we validated our previous results from patients presenting similar phenotypes^6^ (Fig. 1). We hypothesized that the genetic profiles potentially associated with increased and decreased susceptibility to developing tobacco-associated lung adenocarcinoma would be enriched in these populations.^7^, ^8^, ^9^

### Participants

Extreme cases were heavy smokers (≥15 pack-years)^10^ who had developed histologically confirmed lung adenocarcinoma at an early age (≤56 years). We limited inclusion to patients presenting lung adenocarcinoma in order to increase the biological and clinical homogeneity of the study, and because it is the most frequent subtype of lung cancer. We excluded patients presenting tumours harbouring known driver genetic alterations which are not associated with tobacco (i.e., alterations in *EGFR, ALK, ROS1*), because they likely develop through different pathogenic pathways. Extreme cases were lung cancer patients included in the databases from the Oncology and Pulmonary Departments of the Clinica Universidad de Navarra, and the Oncology Department of Hospital General de Valencia.

Extreme controls were heavy smokers (≥15 pack-years) who had not developed lung cancer at an advanced age (≥72 years). Absence of lung cancer was confirmed by chest computerized tomography, since all the extreme controls were participants in a lung cancer screening program implemented in the Pulmonary Department of Clinica Universidad de Navarra.

The thresholds for age and tobacco consumption were those of our previous study^6^ and were established empirically with the aim of selecting from our databases, including over 6600 subjects, those individuals presenting the most extreme phenotypes regarding the individual risk of developing tobacco-associated lung cancer. For this purpose, several combinations of different thresholds for age and tobacco consumption were tested, considering that while more extreme thresholds could define more extreme phenotypes, it would be at the expense of decreased sample size.

The discovery cohort was formed by 50 extreme cases and 50 extreme controls defined by virtually the same criteria for age and tobacco consumption.^6^ There was no overlap between the individuals included in both cohorts.

### Ethics

Samples were provided by the Biobank of the University of Navarra and by the Hospital General Universitario de Valencia, and were processed following standard operating procedures approved by the respective Ethics and Scientific Committees. The protocol was approved by the University Clinic of Navarra Ethics Committee (Reference: 165/2015) and all subjects provided written informed consent.

### DNA extraction and genotyping by WES

Genomic DNA was obtained from peripheral EDTA-blood using the QIAamp DNA Mini Kit (Qiagen Iberia, Madrid, Spain) following the manufacturer's instructions, and was stored at −20 °C until use. Genotyping was performed with a low input protocol using 800 ng of germline DNA after analysis in a TapeStation system (Agilent, Santa Clara, USA) and sequencing with the SureSelect Human All Exon v6 from Agilent at 2 × 150 bp and medium coverage at 200× in a NovaSeq 6000 system (Illumina, San Diego, USA).

### WES variant calling

After quality control (FastQC) and trimming of the reads (trimmomatic),^11^ we performed the read alignment using a BWA-MEM aligner and the GRCh38 human genome assembly as a reference.^12^ The resulting BAM files were processed using an analysis pipeline of variant calling based on GATK best practices.^13^ Several filters were applied such as the variant score normalized by allele depth for a variant (DP <20), the root mean square of the mapping quality of reads across all samples and the strand biases estimated by both Fisher's Exact Test (FS >60) and the Symmetric Odds Ratio Test (SOR >40), among others.

The genetic variants were annotated using ANNOVAR with different databases of genome localization, variant effect prediction, population Single Nucleotide Polymorphisms (SNPs) (ExAC and 1000 Genomes) and clinical association of variants (ACMG, ClinVar, dbSNP and COSMIC).^14^ All the results were integrated and analysed using statistical methods in R/Bioconductor.^15^ For the prioritization of the variants, we selected nonsynonymous variants located in coding regions and in the canonical splice site flanking regions. Types of selected variants included nonsynonymous single nucleotide variants, frameshift insertions and deletions, non-frameshift insertions and deletions and stop gains and losses.

### Association analyses

First, the genetic diversity of the population was assessed using a Principal Component Analysis (PCA) in R/Bioconductor. The first PC only explained 1% of the variability, the barplot of eigenvalues showed a non-structured variation and no subpopulation clusters were observed (data not shown). Considering the homogeneity of the eigenvalues and the low percentage of variability explained by the population diversity in cases and controls, we assumed that we would not have a bias due to subpopulation genome stratification. The variant association analysis was based on the comparison of allele frequencies between the experimental groups, extreme cases and controls, available in the “*allelic*” R package.^16^ This statistical package contains a fast, unbiased and exact allelic test for the association of the variants with the mentioned extreme phenotypes of very high and very low risk of developing tobacco-associated lung adenocarcinoma. The heatmap of the 50 variants with the most significant p-value in this analysis was plotted using the *“ComplexHeatmap”* R package.^17^

In addition, when genes harboured 2 or more variants showing significant differences in their allele frequencies (p < 0.05), a burden analysis at the gene level was performed using the “REBET” R package.^18^ Aggregating those variants by gene, the burden analysis shows if their association is heterogenous or on the contrary the overall association for a gene is more robust than the individual association of its corresponding variants. All possible subsets of the selected variants per each gene were explored and both the gene overall association signal (Meta p) and a separate analysis for high risk (Subset Case Meta-p) and low risk (Subset Control Meta-p) associated variants were performed. The high/low risk association signals were combined using a chi-square test (Subset Meta p).

The selection of disease-associated variants or genes was based on p-values and not on adjusted p-values due to the known low statistical power to study low frequency or rare variants that explain an additional disease risk. Therefore, we validated our results using two different cohorts, as explained in the following section, supporting their replicability.

### Validation of results

#### Validation of individual genetic variants

The genetic variants were considered validated when the same variant in the same gene was identified in both the discovery^6^ and validation cohorts, following the same experimental design. Both results had to be statistically significant (p < 0.05) and show a coherent association with either extreme cases or extreme controls in both cohorts. Variants that were statistically significant but did not show a coherent association with extreme cases or extreme controls in both cohorts were discarded for further analyses. These variants were selected on the basis that if the presence of the same variant increased or decreased in frequency in either cases or controls, there might be a significant role for that specific genetic variant and the effect may not be due to a random event.

#### Validation of variant-rich genes

A gene was considered validated when it harboured significant and coherent variants -either the same or different variants-associated with the phenotypes studied in both cohorts, and the corresponding Subset Case Meta p-value or Subset Control Meta p-value was <0.05. The biological rationale for analysing these variant-rich genes is that they might represent hotspot regions related to the phenotypes studied, as is the case in cancer risk genes, which harbour several different pathogenic variants (e.g.: *BRCA1* and *BRCA2*).

We explored the diseases and biological pathways functionally related to the genes harbouring the variants that were validated using the Reactome Pathway Database.^19^

### Machine learning analysis

#### Machine learning model

The goal of ML models is to predict the phenotype, i.e., case or control, given some input data (referred to as variables or features) related to the genotype. This translates into a supervised learning problem for binary classification, with a positive and a negative class. Without loss of generality, in the following we assume the extreme cases to denote the positive class.

We considered different inputs to train the ML models: presence or absence of relevant variants, or alternatively, the number of variants present in the specific genes of interest. To input the information on the variants on the basis of the “allelic dose” for each individual and genetic variant, we used “0” if no information was available for that variant in that specific individual (no call), “1” to represent the absence of the variant, “2” for a heterozygous variant genotype, and “3” for a homozygous variant.

Considering the sample size, we used the following models: Logistic Regression (LR),^20^ Support Vector Machine Classifier (SVC),^21^ Random Forest (RF)^22^ and Gradient Boosting RF (GbRF).^23^ LR, RF and GbRF fall within the category of soft-classifiers, whereas SVC is a hard-classifier.

The models were implemented using the Python library scikit-learn.^24^

#### Evaluation metrics

As both cohorts were balanced, i.e., they had a similar number of positive and negative instances, we used for model evaluation: accuracy (ratio of all observations correctly predicted by the model), precision (positive predictive value), recall (sensitivity) and F1-score (harmonic mean of precision and recall) metrics. We also considered the Area Under the ROC (Receiver Operating Characteristic) Curve (AUC) to evaluate how efficient a model is in separating positive and negative samples.^25^ Although SVC is not a soft-classifier, it computes a decision function that can be used in a fashion similar to the probabilities for soft-classifiers, permitting the computation of the ROC and hence the AUC.

#### Experimental set-up

The models under consideration (LR, SVC, RF and GbRF) were trained on the discovery cohort and tested on the validation cohort. Each model was trained with different hyperparameters via a grid-search. We used cross-validation (CV) on the discovery cohort to select the best hyperparameters for each model. In CV, the dataset was split into K-folds, with one-fold being left out for model evaluation and the rest used for training. The process was repeated K times, using a different left-out fold each time. Hence, the final evaluation metric for a given model and set of hyperparameters was computed as the average of the accuracies obtained on the left-out folds at each iteration. For each model, the hyperparameters that obtained the highest accuracy were selected. The resulting models were retrained on the entire discovery cohort and tested on the validation cohort.

### Role of funders

The funders had no role in the design and conduct of the study; collection, management, analysis, and interpretation of the data; preparation, review, or approval of the manuscript; and decision to submit the manuscript for publication. None of the authors were paid to write this article by a pharmaceutical company or other agency. The corresponding author states that authors were not precluded from accessing data in the study, and they accept responsibility to submit for publication.

## Results

### Participants

We performed germline WES in samples from 149 individuals, 66 extreme cases and 83 extreme controls (Fig. 1). The mean age for the extreme cases and controls was 50.7 (range 35–56) and 79.1 (74–87) years respectively. Mean tobacco consumption was 34.6 (range 5–99) and 62.3 (30–151) pack-years respectively. The individual characteristics of subjects are provided in Supplementary Table S1.

### Results from the validation cohort

We identified 1133 variants showing significant differences (p < 0.05) [variant association test] in their allelic frequencies between extreme cases and controls in the validation cohort (Supplementary Table S2).

The most significant variant identified in the validation cohort was located in *SELP* (p = 4.74 × 10^−7^) [variant association test]. The 50 top significant variants in the validation cohort are shown in Supplementary Figure S1.

One-hundred and fifty-eight genes included ≥2 significant variants between extreme cases and controls in the validation cohort (range 2–9, Supplementary Table S3). The genes harbouring the most significant variants were *MUC3A* (9 variants, SubsetMeta p = 8.03 × 10^−8^) and *ZNF568* (9 variants, SubsetMeta p = 4.71 × 10^−7^).

### Validation of results

#### Validated individual variants

**Fig. 2:**
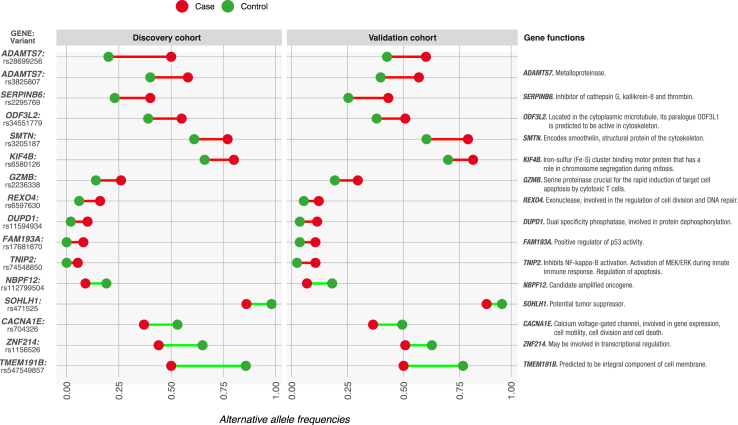
**Validated individual variants.** The figure represents the 16 validated individual variants and the allelic frequencies of each variant in the discovery and the validation cohorts for extreme cases (red dots) and extreme controls (green dots). The differences in allelic frequencies between extreme cases and extreme controls are represented by the red lines (higher allelic frequency in extreme cases) and the green lines (higher allelic frequency in extreme controls). The table also includes selected functions for each gene (obtained from GeneRIF,^26^ GeneCards^27^ and OMIM^28^).

Among the 619 and 1133 significant variants observed respectively in the discovery and validation cohorts, we validated 16 coherent variants. Fig. 2 lists these variants, along with their allelic frequencies as well as selected functions of the genes harbouring them.^26^, ^27^, ^28^ Supplementary Table S4 shows the p-value of each variant in each cohort, as well as additional information from each variant, and the combined p-value for both cohorts [Berger's intersection union test]. The variants presenting the largest differences in allelic frequencies between both extreme phenotypes were located in *ADAMTS7* (2 variants) and *SERPINB6* (higher allelic frequency in extreme cases); and *TMEM191B* and *ZNF214* (higher in extreme controls).

#### Validated genes enriched in significant variants

We searched for genes harbouring significant coherent variants (either the same or different variants) in both cohorts. Table 1 shows these genes along with the p-values [Meta p] in the discovery and validation cohorts and selected functions potentially related to cancer development.^26^, ^27^, ^28^ Supplementary Table S5 shows the specific variants harboured by each gene. In the case of genes with 2 or more significant variants only those genes with all their variants associated with cases (all the corresponding alternative variants are more frequent in cases) or controls (all the alternative variants are more frequent in controls) were considered. In this manner, the genetic implication of the gene in the disease development was clearer.

**Table 1:** Genes harbouring significant variants (same or different variants) in both cohorts.

Gene	Discovery cohort		Validation cohort		Functions
	Number of variants	p-value	Number of variants	p-value	
**Extreme cases: heavy smokers (>15 pack-years) who developed lung adenocarcinoma at early age (≤56 years)**					
*HLA-A*	4	3.460E-07	4	1.673E-06	Antigen presentation.
*ADAMTS7*	2	2.036E-02	2	3.086E-05	Metalloproteinase.
*ANKRD36*	2	3.621E-04	1	4.644E-02	ANKRD36 interacts with the transcription factor YY1 to modulate expression of epithelial sodium channels.
*CRYBG3*	2	1.210E-03	1	4.620E-02	Promotes lung cancer metastasis via activating the eEF1A1/MDM2/MTBP Axis.
*SPINK5*	2	1.971E-03	1	4.448E-02	Tumour suppressor.
*REXO4*	2	2.325E-04	1	4.559E-02	Exonuclease, involved in the regulation of cell division and DNA repair.
*MROH2A*	1	1.248E-02	3	1.464E-05	Encodes a HEAT-domain-containing protein, function yet unknown.
*PDZD7*	1	3.678E-02	2	1.979E-03	Potential oncogene in hepatocellular carcinoma.
*MUC16*	1	2.666E-02	2	1.755E-03	CA125. Thought to provide a barrier, protecting epithelial cells from pathogens and particles. Tumour marker.
*SMTN*	1	2.588E-02	2	1.949E-06	Encodes smoothelin, structural protein of the cytoskeleton.
*TNN*	1	1.248E-02	1	3.560E-02	In tumours, stimulates angiogenesis by elongation, migration and sprouting of endothelial cells.
*PRSS38*	1	2.666E-02	1	4.362E-02	Predicted to enable serine-type endopeptidase activity. Associated diseases include Diamond-Blackfan Anaemia.
*CFAP46*	1	2.088E-02	1	3.655E-02	Part of the central apparatus of the cilium axoneme. Predicted to play a role in cilium movement.
*DUPD1*	1	4.324E-02	1	1.728E-02	Dual specificity phosphatase, involved in protein dephosphorylation.
*DLL3*	1	4.727E-02	1	2.187E-02	Modulation of Notch signalling. Promotes migration and invasion in small-cell lung cancer.
*ODF3L2*	1	2.606E-02	1	4.098E-02	Located in cytoplasmic microtubule, its paralogue ODF3L1 is predicted to be active in the cytoskeleton.
*CABLES2*	1	2.666E-02	1	4.793E-02	Predicted to be involved in cell division and regulation of cell cycle. Proapoptotic factor.
*PCNT*	1	3.946E-02	1	3.956E-02	Interacts with microtubules. Likely important in the function of centrosomes, cytoskeleton and cell-cycle progression.
*FAM193A*	1	5.770E-03	1	1.587E-02	Positive regulator of p53 activity.
*TNIP2*	1	2.647E-02	1	4.203E-03	Inhibits NF-kappa-B activation. Activation of MEK/ERK during innate immune response. Regulation of apoptosis.
*PCDHGA2*	1	9.325E-03	1	3.217E-02	Potential calcium-dependent cell-adhesion protein.
*KIF4B*	1	4.159E-02	1	3.337E-02	Iron-sulphur (Fe-S) cluster binding motor protein that has a role in chromosome segregation during mitosis.
*SERPINB6*	1	1.457E-02	1	1.871E-03	Inhibitor of cathepsin G, kallikrein-8 and thrombin.
**Extreme controls: heavy smokers (>15 pack-years) who did not develop lung adenocarcinoma at advanced age (≥72 years)**					
*PLIN4*	2	5.976E-04	2	1.615E-03	Lipid droplet metabolism.
*ZNF214*	2	6.689E-05	1	3.643E-02	May be involved in transcriptional regulation.
*NBPF12*	1	4.396E-02	5	7.976E-09	Candidate amplified oncogene.
*KRT18*	1	2.783E-02	2	2.787E-03	Cytoskeleton protein. Consistently expressed in many tumour types, especially adenocarcinomas.
*DNAH11*	1	2.417E-02	2	2.818E-05	Microtubule-dependent motor ATPase. Reported to be involved in the movement of respiratory cilia.
*CACNA1E*	1	3.193E-02	1	2.527E-02	Calcium voltage-gated channel, involved in gene expression, cell motility, cell division and cell death.
*PITRM1*	1	4.338E-02	1	3.437E-02	ATP-dependent metalloprotease that degrades post-cleavage mitochondrial transit peptides.
*TMEM191B*	1	3.195E-02	1	4.066E-02	Predicted to be an integral component of membrane.
*WRN*	1	3.365E-02	1	6.716E-03	Helicase involved in DNA repair, maintenance of genome stability and telomeres, replication and transcription.
*SOHLH1*	1	3.362E-03	1	3.462E-02	Potential tumour suppressor.

The table represents the number of variants per gene in extreme cases and controls and in the discovery and validation cohorts, along with the respective p-values. In the case of genes with several genetic variants the meta p value from the “REBET” R package is shown, while in the case of single variants, the individual p-values of the “allelic” R package are shown. See Materials and Methods section for further details. The table also includes selected functions for each gene (obtained from GeneRIF,^26^ GeneCards^27^ and OMIM^28^).

These genes might represent hotspot areas related to the phenotypes studied, especially those harbouring more than one variant. Interestingly, *HLA-A* harboured the largest number of significant variants in extreme cases from the discovery and validation cohorts, achieving p-values of 3.46 × 10^−7^ and 1.67 × 10^−6^ respectively [Meta p]. *HLA-A* has a major role in antigen presentation to CD8 T-cells, and possibly in cancer immune surveillance. Since tobacco has the potential to induce tumour neoantigens, these findings warrant further study.

We performed a Reactome Pathway Database analysis of the 33 variant -enriched genes. The most significant biological pathways associated with these genes were related to immune response, including antigen processing and presentation and cytokine and interferon signalling, achieving highly significant p-values [hypergeometric test] in the overall analysis and in the False Discovery Rate (FDR) (Supplementary Table S6 and Supplementary Figure S2).

### Machine learning model

We performed ML analysis using as input the genotype information of the 16 significant reported variants and the number of variants for each of the 33 validated genes. In all cases models were tuned through the CV process using the discovery cohort. The hyperparameters and values taken into consideration for each model are presented on Supplementary Table S7.

**Fig. 3:**
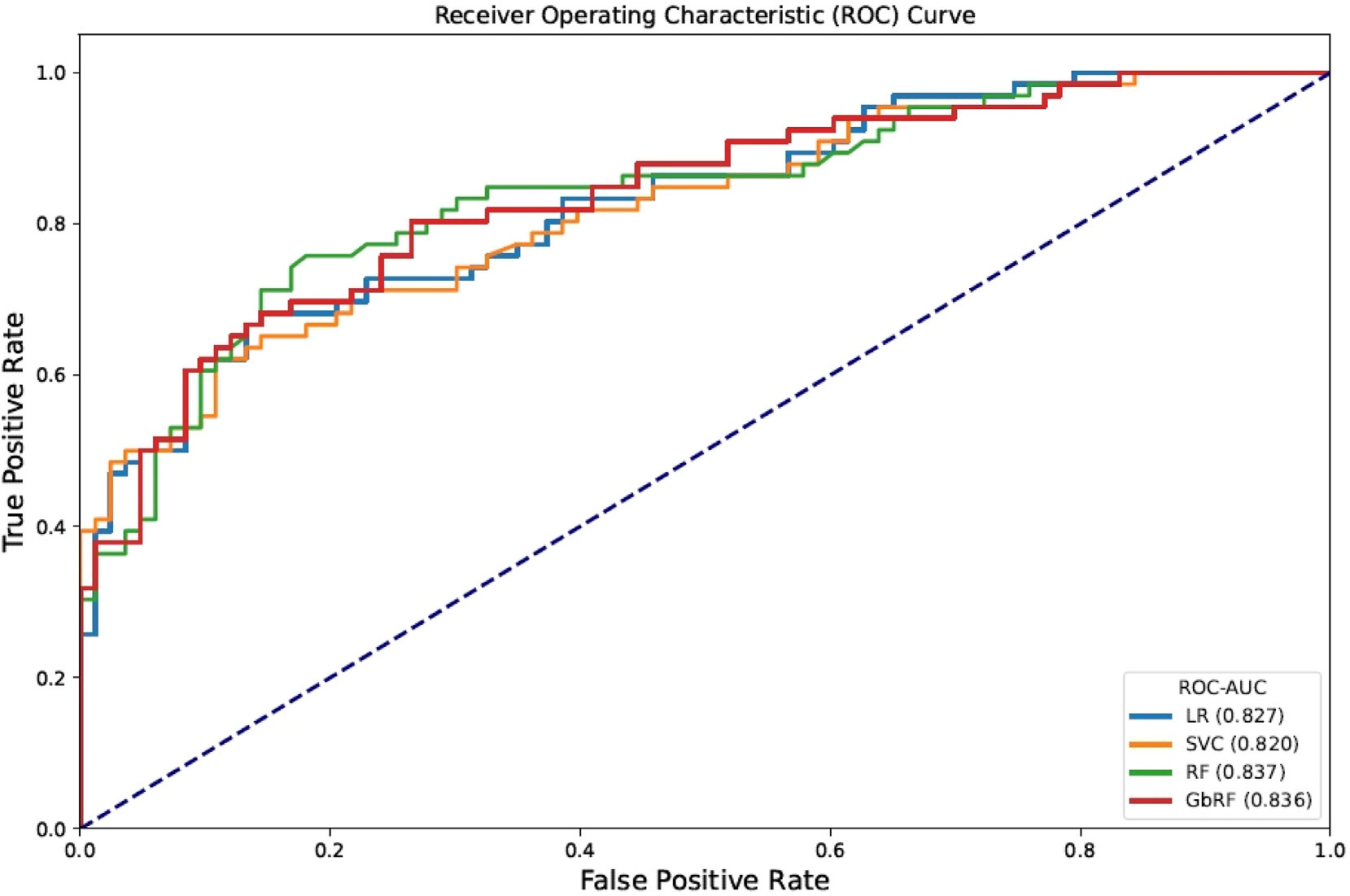
Receiver Operating Characteristic-Area Under the Curves (ROC-AUC) and values obtained for the validation cohort using the four ML models considered, with optimized hyperparameters, using as input the genotype of the 16 significant variants. LR: Logistic Regression. SVC: Support Vector Machine Classifier. RF: Random Forest. GbRF: Gradient Boosting Random Forest.

The best results were obtained with the 16 significant variants as input. Table 2 shows the accuracy and ROC-AUC values obtained on the discovery cohort, via CV, for each of the models considered. For each model results are shown with the best set of hyperparameters. All models achieved similar results, with accuracy values ranging from 0.74 to 0.78, and ROC-AUC values from 0.86 to 0.89 on the discovery cohort. When evaluating the models on the validation cohort, GbRF offered the best performance: accuracy above 0.76 and ROC-AUC above 0.83. On the validation cohort, more than 77% of the extreme cases were correctly predicted by all models (recall), while the rate of extreme controls correctly classified ranged from 62% with RF and GbRF to 67% with LR (Supplementary Figure S3). In terms of recall, precision and F1-score, GbRF obtained the best results (Table 2). Fig. 3 shows the ROC-AUC achieved by the four considered ML models for the validation cohort, using as input the genotype of the 16 selected variants.

**Table 2:** Performance metrics for the Machine Learning (ML) models.

Models	Discovery cohort (CV)		Validation cohort				
	Accuracy	ROC-AUC	Accuracy	ROC-AUC	Precision	Recall	F1-score
LR	0.780	0.896	0.691	0.826	0.622	0.773	0.689
SVC	0.780	0.868	0.718	0.820	0.654	0.773	0.708
RF	0.740	0.885	0.752	0.837	0.688	0.803	0.741
GbRF	0.740	0.864	0.765	0.836	0.707	0.803	0.752

Performance metrics obtained on the discovery cohort via CV for the four ML models considered (with optimized hyperparameters), as well as on the validation cohort (not seen during training), using as input the genotype of the 16 selected variants. GbRF obtained the best results when using as input the genotype of the 16 significant variants. CV: Cross-validation. ML: Machine Learning. ROC-AUC: Receiver Operating Characteristic-Area Under the Curve. LR: Logistic Regression. SVC: Support Vector Machine Classifier. GbRF: Gradient Boosting Random Forest. RF: Random Forest.

## Discussion

Tobacco is the most relevant risk factor for the development of lung cancer. However, remarkable differences in the susceptibility of different individuals to developing tobacco-associated lung cancer exist. Here, we validated the characterization of individuals presenting extremely high and low risk of developing tobacco-associated lung adenocarcinoma by WES germline analysis and we developed a ML model based on our findings. High-risk subjects (extreme cases) were patients who had been heavy smokers and had developed lung cancer at a young age; and low-risk subjects (extreme controls) were also heavy smokers but who had not developed lung cancer at an advanced age. These individuals likely represent real life models of extreme sensitivity and resistance to the carcinogenic effects of tobacco. We limited recruitment of extreme cases to lung adenocarcinoma patients, and we excluded patients harbouring known driver genetic alterations not related to tobacco, to increase homogeneity.

We validated individual variants presenting significantly different allelic frequencies as well as genes harbouring the same or different significant variants in the discovery and validation cohorts. Notably, the function of many genes harbouring validated variants is related to carcinogenesis, including candidate oncogenes, tumour-suppressor genes, and genes involved in DNA repair, maintenance of genomic stability, HLA-mediated antigen presentation and regulation of proliferation, migration, apoptosis and immune response, among others (Fig. 2 and Table 1). Indeed, the extreme phenotypes that we characterized may be related to different molecular mechanisms.

We explored the ability of ML models to classify the study subjects. The GbRF model, using the genotype of the 16 validated individual variants, offered the best performance in terms of accuracy, ROC-AUC value, precision and F1-score. We acknowledge that the trained models are not able to fully characterize the two populations with the information provided. More information about the genotype or information from larger cohorts may be needed to boost the performance of the ML models. Nevertheless, these results show that ML may be a useful tool to learn complex patterns and characterize these clinically relevant populations based on genotype data.

The search for genetic variants associated with cancer risk is limited by their low frequency and variable penetrance, as seen in pathogenic hereditary cancer variants. These characteristics hamper their identification and validation, which mainly relies on global networks of certified genetic and clinical research institutions. Selection of individuals presenting phenotypes of extremely high cancer risk may facilitate the identification of these variants, as seen with pathogenic variants in *TP53* and *BRCA*, discovered through the study of individuals presenting early-onset cancer and familial aggregation.^29^, ^30^, ^31^ Of note, most known high-risk cancer variants have been discovered through association with familial aggregation, while our strategy assesses high-risk cancer variants outside this framework.

To date, the field of cancer protective genetic variation remains largely unexplored. Yet, protective genetic variants have been reported for other diseases. For example, specific genetic variants in the gene encoding the chemokine coreceptor CCR5 confer protection from certain strains of the Human Immunodeficiency Virus^32^^,^^33^; and nonsense mutations in *PCSK9* are associated with decreased levels of serum low density lipoproteins and reduced risk of cardiovascular disease,^34^ among others. Interestingly, these protective genetic variants were discovered by studying individuals presenting extreme phenotypes, thus further supporting the use of this strategy.^7^, ^8^, ^9^

Tobacco is the most relevant risk factor for developing cancer. Consequently, the identification of genetic profiles associated with increased intrinsic susceptibility to or protection from its carcinogenic effects could have major implications for public health, such as allowing the identification of high-risk individuals for implementation of tobacco cessation and cancer screening programs. It could also improve our understanding of carcinogenesis and physiological protection against cancer development, guiding new preventive, diagnostic and therapeutic approaches.

The main limitation of our study is that, despite being the largest series of its kind, additional studies will be required to further characterize these relevant phenotypes. Indeed, our study provides the rationale to further develop this strategy in the setting of larger databases, optimizing our already stringent selection criteria. From a clinical perspective, this could be achieved by selecting individuals presenting more extreme ages and/or higher exposure to tobacco (i.e.,: higher consumption, shorter tobacco cessation intervals, inclusion of active smokers, etc.). Additional studies following this strategy will also be required to address the potential impact of other relevant variables, such as gender or ethnicity, as well as the effect of potential confounding factors.

From the perspective of the clinical and pathological characteristics of tumours, the selection criteria could be improved by studying patients presenting similar dissemination patterns or more specific tumour molecular profiles (e.g.: either *KRAS* positive or negative tumours).

The stringent selection criteria do not preclude the possibility that extreme controls may develop lung cancer in the future. Yet, the large differences between the mean ages between both groups clearly define very distinct subpopulations. The possibility that lung adenocarcinoma may be induced by causes other than tobacco in some cases, must also be considered. The biological relevance of the variants identified must be confirmed in functional studies.

Further development of this methodology should also address the optimal definition of extreme phenotypes, establishing the optimal thresholds for age and tobacco consumption, as well as considering additional variables related to the risk of developing tobacco-induced lung cancer. Other aspects to develop include the determination of the optimal sample size and the application of alternative designs, such as extreme phenotype vs. control population studies,^35^ which may broaden the applicability of results to general, non-extreme populations. Our strategy may also be applied to other tumours, either related to tobacco or to other well-characterized risk factors. The study of patients developing tumours presenting an even closer relationship with tobacco, such as squamous lung carcinoma, or small cell lung carcinoma, could yield relevant results. Here, we chose lung adenocarcinoma due to its higher frequency. Finally, our study could be improved by analysing additional biological variables, such as the non-coding part of the genome, which could be evaluated through Whole Genome Sequencing, or using different strategies in the analysis, such as focusing on variants that are exclusive to either the extreme cases or extreme controls, constructing polygenic risk scores (PRSs) or other models of combination of the genetic diversity to strengthen the power of discrimination between groups.

In conclusion, we have validated the germline profile of individuals presenting extreme phenotypes of very high and very low risk for developing tobacco-associated lung cancer. This strategy may allow individuals presenting these clinically relevant phenotypes to be identified, and the underlying biological mechanisms to be understood.

## Contributors

JLPG and APG conceptualised, designed and supervised the study. JLPG acquired the funding. MPA, MO, AG, CC, EJL, MNA, MFS, IM, JJZ, JPT, JPF, GB, LS, LMM and JLPG contributed the study samples and clinical data. APG and GAA analysed the samples. EG and APG performed the Bioinformatics. IO, MH, JJFS and NVZ. performed the Machine learning models. JLPG, APG, EG, IO, MH, MPA, MO, JJFS, NVZ, GAA, ITU, AG, CC, EJL, MNA, MFS, IM, ME, JPF, GB, LS, RP and LMM interpreted the data. JLPG, APG, EG, IO, MH, JJFS, GAA, ITU, MFS, IM, ME, JJZ, JPT, GB, LS, RP and LMM drafted the manuscript. JLPG, AP, EG and IO have accessed and verified the underlying data. All authors read and approved the final version of the manuscript before submission.

## Data sharing statement

Genomic data from study participants are available upon reasonable request from the corresponding author.

## Declaration of interests

MFS reports grants from Bristol Myers Squibb and Roche, as well as honoraria from lectures and advisory from MSD, BMS and Numab; and travel support from Roche, Astra-Zeneca and BMS.

IM reports receiving commercial research grants from AstraZeneca, BMS, Highlight Therapeutics, Alligator, Pfizer Genmab and Roche; has received speakers bureau honoraria from MSD; and is a consultant or advisory board member for BMS, Roche, AstraZeneca, Genmab, Pharmamar, F-Star, Bioncotech, Bayer, Numab, Pieris, Gossamer, Alligator and Merck Serono.

JJZ declares consultancy and advisory Board from American Heart Technologies and Median Technologies.

GB declares honoraria from lectures and advisory from General Electric, Siemens Healthineers; educational activities for General Electric, Siemens Healthineers, Bayer; and institutional research grants from Siemens Healthineers, Guerbet.

LMS reports a role as scientific advisor for Sabartech, Serum, Astra Zeneca, Roche, MSD, and Median technologies, and has received honoraria as a speaker from Astra Zeneca, GSK, Roche, Menarini, and Chiesi.

LMM declares research grants from Astra-Zeneca, BMS, Serum Detect Inc. and Pharmamar; speaker fee from Astra Zeneca; participation in advisory boards from Serum Detect Inc; and has a Licenced patent co-holder from AMADIX.

JLPG declares research grants and support from Astellas, Amgen, BMS, MSD, Novartis, Roche, Seattle Genetics; participates in advisory boards for Astellas, BMS, Ipsen, MSD, Roche, Seattle Genetics; and travel support from BMS, MSD, Roche.

## Appendix A Supplementary data

Supplementary Tables Supplementary Table S1: Individual characteristics of subjects included in the study. F: female. M: male. Supplementary Table S2: Significant variants identified in the validation cohort. Supplementary Table S3: Genes including >2 significant variants between extreme cases and controls in the validation cohort. Supplementary Table S4: Validated individual variants. Supplementary Table S5: Variants in genes harboring significant variants (same and different variants). Supplementary Table S6: Reactome Pathway Database analysis of pathways related to the validated genes enriched in significant variants. Supplementary Table S7: Hyperparameters considered for each model, together with the values tested through the CV process. Values in bold correspond to those obtaining the best results when using as input the genotype of the 16 selected variants.
