# Supplementary material for: Whole exome sequencing and machine learning germline analysis of individuals presenting with extreme phenotypes of high and low risk of developing tobacco-associated lung adenocarcinoma
Source: eBioMedicine. 2024 Mar 13;102:105048. doi: 10.1016/j.ebiom.2024.105048 (PMC10955643; doi:10.1016/j.ebiom.2024.105048)

Supplementary Figure 1: Representation of the 50 variants with the most significantly different allelic frequencies between individuals presenting extreme phenotypes of high and low risk of developing tobacco-induced lung adenocarcinoma in the validation cohort.

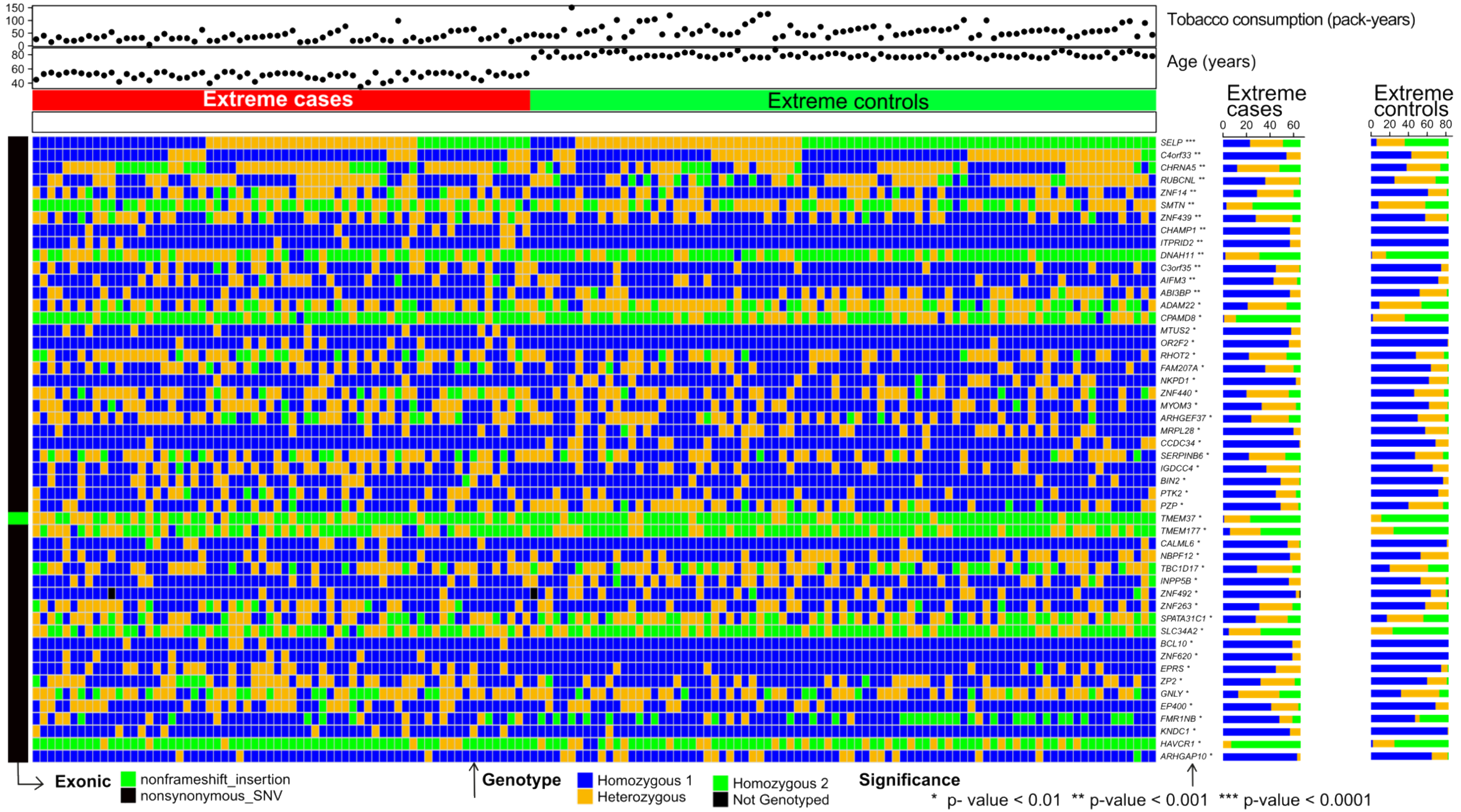

Supplementary Figure 2: Reactome Pathway Database analysis of pathways related to the validated genes enriched in significant variants.

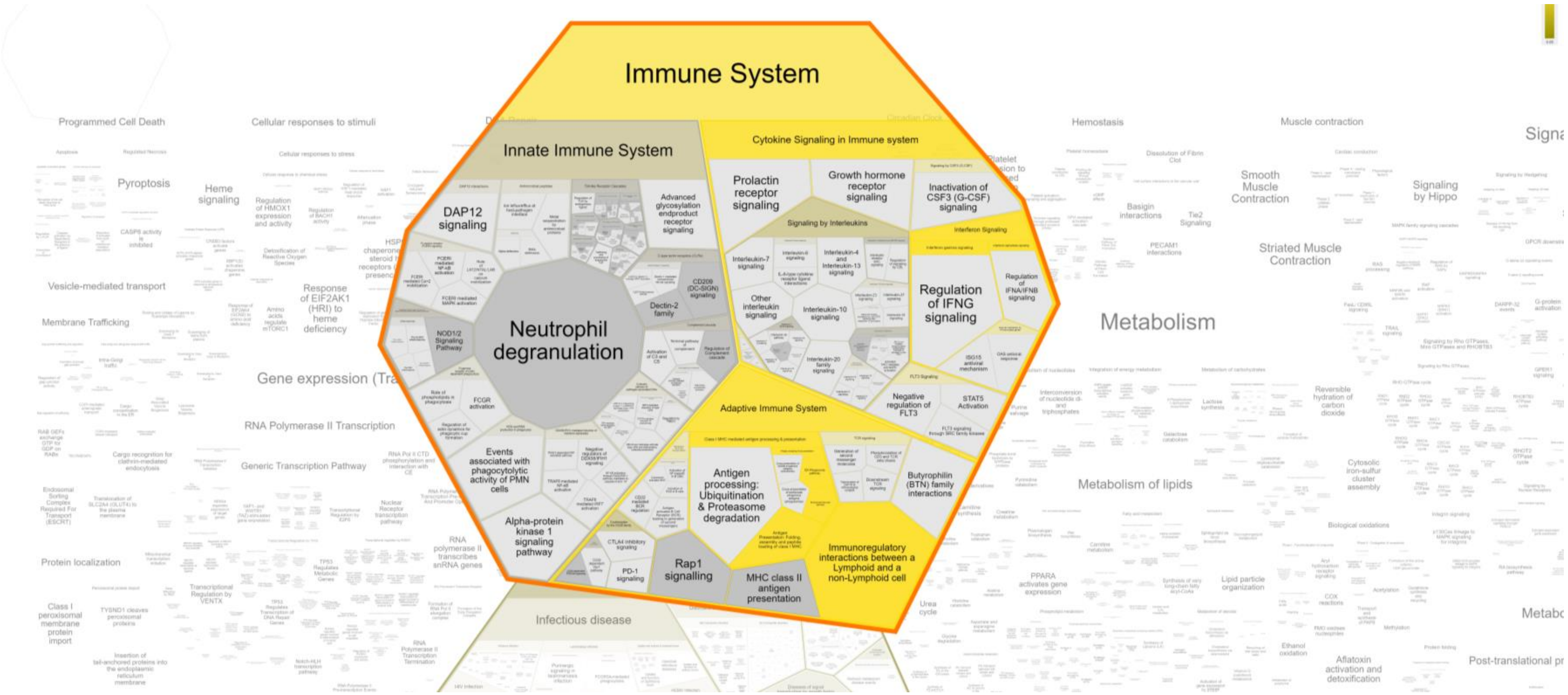

Supplementary Figure 3: Confusion matrices obtained by LR, SVC, RF and GbRF (A-D), respectively, on the validation cohort, with the obtained performance metrics (accuracy, precision, recall, and F1-score). LR: Logistic Regression. SVC: Support Vector Machine Classifier. GbRF: Gradient Boosting Random Forest. RF: Random Forest.

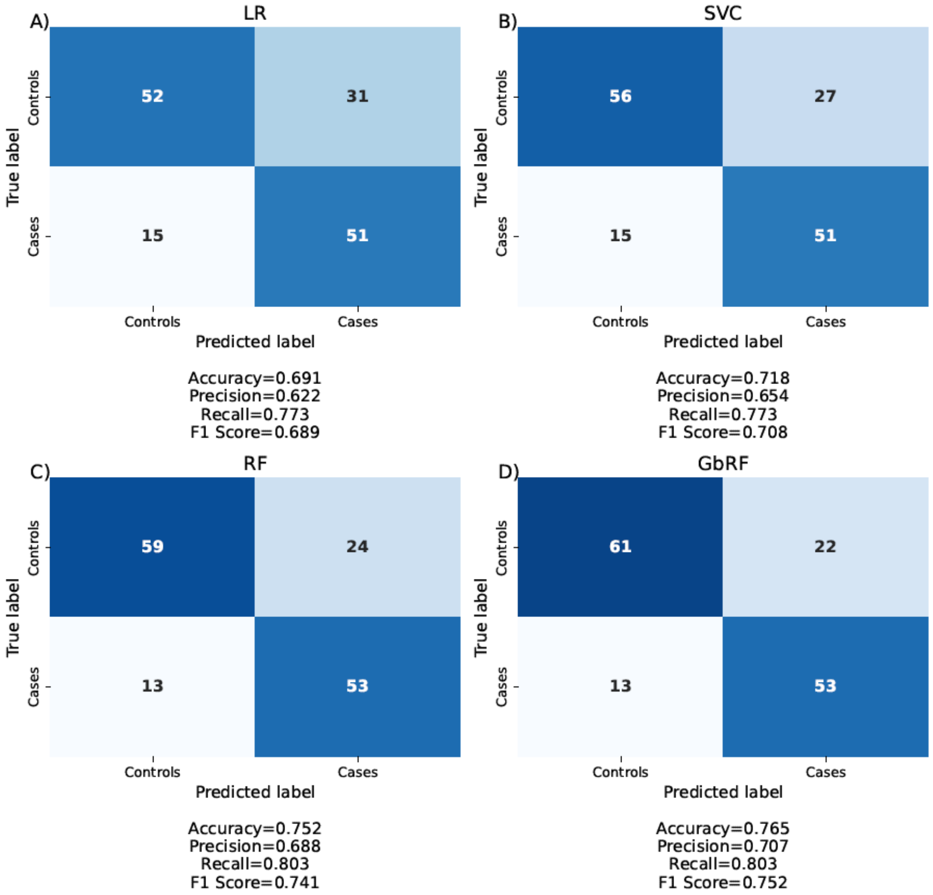

Supplement: Supplementary Figures [file mmc2.pdf]
